# The Capital of the Central Funds

**Published:** 1920-08-14

**Authors:** 


### The Hospital

The Workers' Newspaper of Administrative Medicine and Institutional Life, Administration, National Insurance and Health.

No. 1784, Vol. LXVIII. SATURDAY, AUGUST 14, 1920. PRICE TWOPENCE

### THE CAPITAL OF THE CENTRAL FUNDS.

The meeting of the Council of the Metropolitan Hospital Sunday Fund last week was noteworthy for two reasons, only one of which was the distribution of £110,000. The second point, perhaps even more ultimately important, was the suggestion that the Fund should realise some of its capital to increase the grants made to hospitals and dispensaries this year. Since the total distribution surpassed all that those in touch with the Fund had expected, we are glad that this achievement is undimmed by any such desperate expedient. Moreover, the suggestion had already been considered and disposed of for the present at a special meeting of the Distribution Committee which was held on July 6. The committee decided not to follow the precedent created earlier in the year by the emergency grant which King Edward's Hospital Fund had distributed by realising some of its capital. In view of the sum distributed last week, the committee will have been pleased at its own decision, especially since the latest advocates of an encroachment upon the Fund's capital accompanied their request by a very gloomy forecast of the hospitals' immediate future. This raises the general question whether the capital of the Central Funds should be regarded as sacred or whether it should be at the beck of institutions in urgent need of money.

We do not think the principle can be regarded as settled merely because the King's Fund realised some of its capital to make an "emergency grant" this year. No rule is so strict that at some time it may not be broken in the letter. But to say this is very different from proposing that the capital of one after another of the Central Funds should hastily be realised. The proposal involves immediate and ultimate difficulties, and we hope that the former will suffice to show the danger attaching to the full realisation of the plan. In the first place, capital can hardly be realised for the benefit of one hospital without creating jealousy, perhaps rewarding inefficiency unfairly, and raising a general clamour which may lead to the extinction of the Fund. For there can be no doubt that the donors to the great Funds act from motives somewhat different to those which inspire subscriptions and even legacies to particular hospitals. The donors of the latter wish to support an institution which they favour, and know that in an emergency their gift of capital may have to be used to pay current accounts. But donors to the great Funds are more interested in the voluntary system than in particular hospitals, and believe that their money will be spent to the best advantage through the agency of the distribution committees of the Funds. These donors respect the power of the purse, and regard the Funds as Trustees of the voluntary system who inspect as well as give, and maintain a standard of general efficiency which the mere emulation of one hospital with another is not sufficient to produce. We do not believe that these benefactors would be other than dismayed if they thought that the capital to which they had contributed would be dispersed, and are convinced that they gave upon a tacit understanding that the Funds exist to maintain, not to rescue, the voluntary hospitals. The proof of this contention would be found if the dispersion of their capital by the three Funds led to a falling off in their legacies; and, if it did, a blow would have been struck at the Funds themselves from which they would not easily recover. Beside this the sale of capital to meet the present emergency would involve a positive loss through the depreciation of many of the Funds' investments, and, in the long run, they would be permanently weakened thereby. It is natural to big institutions in severe straits to seek to lay hands on any money that may seem available, but such institutions should think twice before they endanger the existence of Funds which are the chief supports of an entire system.

Another matter of equal importance is the position of Funds in regard to the Ministry of Health. So long as their capital remains untouched they are great corporations, the supporters and controllers of the London hospitals, and to them the Ministry can talk in any proposals either to distribute a Government grant or to act as intermediary between the Ministry and the hospitals.

Once their capital were dispersed they would lose their character and prestige, and, if the prophecy of the alarmists is fulfilled, in a year or two's time the hospitals would be where they are now, except that the Funds would be no longer in existence to help them. We believe, in short, that the proposal would strike a more severe blow at the voluntary system than the present difficulties can possibly do. So long as the Funds remain, the voluntary system is sufficiently entrenched to survive and influence any subsequent modifications. Were their capital dispersed, the chief support of the system would have been removed, and the voluntary system as such would have no body or centre of influence.

In conclusion, it appears that the proposal is favoured by those hospitals which have cultivated no regular source of income except that of the personal appeal. The success of this was proved during the war, but the Red Cross Society itself is finding that the force of these appeals is transient, and is relying on special weeks and similar instances of methodical organisation to raise the large sums which, during the war, the joint societies received merely for the asking. Without committing ourselves to Sir Napier Burnett's belief that the method of personal appeal is "dead" everywhere, we are sure that methodical collections, especially in London, must be organised much more thoroughly than in the past. For these reasons we applaud the decision of the Sunday Fund not to realise its capital, because so long as it remains intact the voluntary system has a guarantee of endurance, which it would be suicide to throw away. So long as the great Funds remain, the London hospitals will be respected by the State. Once the Funds are weakened the Government will feel that it can do what it likes with them. For the Funds are a means of permanent support, and it is those permanent means which most need strengthening at present.

